# Comparison of module detection algorithms in protein networks and investigation of the biological meaning of predicted modules

**DOI:** 10.1186/s12859-016-0979-8

**Published:** 2016-03-18

**Authors:** Shailesh Tripathi, Salissou Moutari, Matthias Dehmer, Frank Emmert-Streib

**Affiliations:** Predictive Medicine and Analytics Lab, Department of Signal Processing, Tampere University of Technology, Tampere, Finland; Centre for Statistical Science and Operational Research, School of Mathematics and Physics, Queen’s University Belfast, Belfast, UK; Institute for Theoretical Informatics, Mathematics and Operations Research, Department of Computer Science, Universität der Bundeswehr München, Munich, Germany; Institute of Biosciences and Medical Technology, Tampere, Finland

**Keywords:** Module detection algorithms, Protein networks, Biological processes

## Abstract

**Background:**

It is generally acknowledged that a functional understanding of a biological system can only be obtained by an understanding of the collective of molecular interactions in form of biological networks. Protein networks are one particular network type of special importance, because proteins form the functional base units of every biological cell. On a mesoscopic level of protein networks, modules are of significant importance because these building blocks may be the next elementary functional level above individual proteins allowing to gain insight into fundamental organizational principles of biological cells.

**Results:**

In this paper, we provide a comparative analysis of five popular and four novel module detection algorithms. We study these module prediction methods for simulated benchmark networks as well as 10 biological protein interaction networks (PINs). A particular focus of our analysis is placed on the biological meaning of the predicted modules by utilizing the Gene Ontology (GO) database as gold standard for the definition of biological processes. Furthermore, we investigate the robustness of the results by perturbing the PINs simulating in this way our incomplete knowledge of protein networks.

**Conclusions:**

Overall, our study reveals that there is a large heterogeneity among the different module prediction algorithms if one zooms-in the biological level of biological processes in the form of GO terms and all methods are severely affected by a slight perturbation of the networks. However, we also find pathways that are enriched in multiple modules, which could provide important information about the hierarchical organization of the system.

## Background

The biological function on the molecular level emerges from the complex interaction of biological entities of a cell [1, 2]. Specifically, different types of molecules, e.g., proteins, metabolites, miRNA or tiRNA, can interact in many various ways with each other in dependence on the tissue type and the environmental condition of an organism. The interactions among biological molecules can be broadly categorized into three types of networks: metabolic networks, transcriptional regulatory networks and protein interaction networks [3–6]. These networks need to be inferred from experimental observations generated by different high-throughput platforms, including Next-Generation Sequencing (NGS), proteomics and microarrays.

Nowadays, it is generally accepted that biological networks are not randomly connected but follow certain structural patterns that give rise to (I) a scale-free topology, (II) a hierarchical organization and (III) a modular structure [7–12]. Especially modularity is one of the most important features of biological networks, because it suggests that nodes, which are tightly connected with each other as a community, are most likely to be a part of the same biological function or pathway. This may also be reflected in the evolution of the organisms [8, 13–15]. As a complicating factor, in reality, these pathways are not discrete, but each gene may take part in multiple biological functions, and therefore can be a part of multiple communities. Hence, a biological network with a modular structure can contain multiple overlapping communities, which might also contribute to the fact that biological networks are robust [16, 17].

For protein interaction networks (PINs) it is known that there are two types of modular structure that are of significant importance. These modules can be either formed by protein complexes or dynamic functional units [18]. Also the modules in PINs of different species have been explained as the efficient functioning of a cell and the basis of evolution in order to adapt the changes to the environment quickly [19, 20]. In [21] the existence of two further types of structural components of modules in protein networks has been revealed, which have been termed core components and ring components. The core components are more conserved and perform key biological functions, while the ring components performs certain specialized functions under particular circumstances potentially triggered by environmental changes. Furthermore, several methods have been developed to identify and integrate protein networks along with gene expression or other datasets such as disease-gene association to identify the functional activity of modules in different disease conditions [22–25]. Finally, in [26] the algorithm *ClusterONE* has been developed to identify overlapping nodes in modules in protein networks. These examples demonstrate that any systems-based analysis on the genomic level is incomplete without a network understanding of interactions on the molecular level.

Our study has four major objectives. The first objective of our study is to compare community detection algorithms for benchmark networks as well as 10 protein interaction networks. Second, we provide an in depth analysis of the biological meaning of the predicted networks across a variety of different biological aspects. Third, due to the fact that all PINs are inferred from experimental data they carry a certain uncertainty with respect to the correctness of the inferred interactions. For this reason, we are performing a robustness analysis of the predicted modules by perturbing the PINs by edge deletions. Finally, we investigate overlapping pathways that may form functional bridges between more specialized modules.

For the community detection analysis, we are using the 5 most popular module detection algorithms, fast-greedy [27], walktrap [28], label propagation [29], spinglass [30] and multi-level community [31], that have been developed for application to large networks and propose in addition 4 correlation-based module prediction methods. Briefly, for our approaches, we assign weights to each pair of nodes depending on the distance between them in the network and utilize this for the module prediction. This provides competitive modularity measures for artificial and biological networks in comparison to other community detection algorithms. The details about all measure will be given in the Methods section.

Typically, for large real networks there is only limited information available about the true module structure within these networks because of our lack of understanding of the underlying phenomena. However, for protein networks we can make use of the Gene Ontology (GO) database [32], which provides a comprehensive overview of thousands of biological processes in a variety of different organisms. Utilizing this information allows a biologically meaningfully assessment of the predicted modules. Specifically, in our analysis, we use protein networks of 10 different species to investigate the modularity predicted by the different community detection algorithms.

This paper is organized as follows. In the next section, we describe all methods, measures and data sets used for our analysis, including a description of the protein interaction networks. In the Results section, we present our numerical findings and this paper finishes with the Conclusions section summarizing and discussing our results.

## Methods

### Modularity

The module detection algorithms studied in this paper, optimize the modularity in a network. The measure for the modularity has been introduced in [27, 33] and is defined as follows.

$$ Q = \Sigma_{i} \left(e_{ii} - (a_{i})^{2}\right) $$

where *e*_*ij*_ is a fraction of edges between communities *i* and *j*,

$$e_{ij} = \frac{1}{2m} \Sigma_{v \in i, w \in j} A_{vw} $$

*A*_*vw*_ is the adjacency matrix element between *v* and *w* and *a*_*i*_ is the fraction of edges which is connected to the nodes in community *i*, i.e.,

$$a_{i} = \frac{1}{2m}\Sigma_{v \in i} k_{v} $$

Here *k*_*v*_ is a degree of node *v*∈*i*.

### Fast-greedy algorithm

This method was proposed in [27]. The algorithm starts with the assumption that each individual node is an independent community and assigns modularity score, *ΔQ*_*ij*_, to each pair of nodes, and *a*_*i*_ for each community. The *ΔQ*_*ij*_ and *a*_*i*_ are defined as follows:

$$\begin{array}{*{20}l}  \Delta Q_{ij} &= \left\{ \begin{array}{ll} \frac{1}{2m}- \frac{k_{i} k_{j}}{(2m)^{2}} &\quad \text{if i, j are connected;}\\ &\quad\text{m is the total number of edges}\\ 0 &\quad \text{otherwise}. \end{array}\right.\\ a_{i}& = \frac{k_{i}}{2m} \end{array} $$

The algorithm starts by calculating *ΔQ*_*ij*_. Then it merges the two communities for which *ΔQ*_*ij*_ is largest. After that, it updates *ΔQ* and *a*_*i*_ for each community and repeats all steps until all communities are merged into one community. When two communities, *i* and *j* are merged the *ΔQ* is updated as follows:

$$ \Delta Q = e_{ij} +e_{ji} - 2a_{i}a_{j} $$

### Walktrap algorithm

This method was proposed in [28]. The algorithm starts with the assumption that if two vertices, *i* and *j*, are in same community, then the random walk of length *t* from *i* and *j* to the nodes of other communities would be similar, $P_{ik}^{t} \sim P_{jk}^{t}$. The random walk starting at vertex *i* to *j* through a path of length *t* is described as follows:

$$ \forall i, \, lim \, t \rightarrow +\infty \, P_{ij}^{t} = \frac{d(i)}{\Sigma_{k} d(k)} $$

where *d*(*i*) is the degree of vertex *i*.

In the first step of the algorithm, all nodes are considered as individual communities. In the second step, the two closest communities are merged based on the distance between them, and the community structure is updated. Then the second step is repeated until all communities are merged into one community.

The distance between communities is calculated as follows. Suppose there are *C*=*C*_1_,*C*_2_…*C*_*k*_ communities in the network.

$$ \sigma_{k} =\frac{1}{n} \Sigma_{{C_{k}}\in{C}}\Sigma_{{i \in {C_{k}}}} r^{2}_{i,C_{k}} $$

*σ*_*k*_ is a mean square distance between two communities. The *r* is defined as follows:

$$r_{{C_{i}}{C_{j}}} = \sqrt{\Sigma_{k=1}^{n} \frac{\left(P^{t}_{C_{i}k}-P^{t}_{C_{j}k}\right)^{2}}{d(k)}} $$

### Label propagation algorithm

This method was proposed in [29]. In this approach, a node *x* chooses to community to which the maximum numbers of its neighbours belong to. There are following steps to identify communities in the network.

1. Assign a unique label to each node.
2. Order nodes randomly.
3. label the selected node with the same label which is in maximum number in its neighbourhood.
4. If all the nodes have the same label, which is in maximum number in their neighbourhood, then stop the algorithm, otherwise repeat step 3.

### Spinglass community algorithm

This method was proposed in [30]. In this approach the community detection is mapped to finding the ground state of an infinite ranged Potts spin glass model, by combining the information from both present and missing links, where the clusters are represented as the number of occupied spin states. In the Spinglass algorithm, existing edges within a community and non-existing edges between communities are rewarded while the edges which are not present in the community and edges between communities are penalized.

### Multi-level community algorithm

This method was proposed in [31]. This algorithm is divided into two phases. In the first phase, all nodes are considered as independent communities. Then communities are merged into a larger community if the modularity of the network increase. The first phase is stopped if there is no further increase in the modularity. In the second phase each community is represented in the form of a node and edges between and within communities are replaced by weighted-edges. The number of edges between two nodes (communities) are replaced by a single weighted edge and all the edges in a community are replaced by a self-connecting weighted edge. After the construction of a new weighted network, first phase is repeated to obtain an improvement in modularity. These two phases are iterated until there is no further improvement in the modularity of the network.

### Correlation based hierarchical clustering

In this approach, we start with the assumption that if two nodes are the part of same community then their shortest path distance to all other nodes are positively correlated. We first calculate the shortest path distance, *S*(*G*) for a graph *G*, between all pairs of nodes an calculate correlation between each pair of nodes. Here we provide some shortest path based measures to calculate correlation between pairs of nodes. Let *S*(*G*) is the shortest path distance matrix, and the correlation matrix is *ρ*(*S*(*G*)), then the distance between each pair of node is described as follows:

$$ D_{sp} = 1 - \rho(S(G)) $$

The second measure for correlation is described as follows: Let *A* and *S*(*G*) are adjacency and shortest distance matrix for a graph *G*, then the weight matrix of pairs of nodes.

$$ M = A \times S(G) $$

*M* is an asymmetric weight matrix where each row represent nodes and columns represent weights between each other. If the nodes are from same community then their weights w.r.t other nodes are strongly correlated. The distance matrix is defined as follows:

$$ D_{M} = 1 - \rho(M) $$

We use these two different distance measures for hierarchical clustering (ward algorithm). To get an optimal number of cluster we use modularity measure by newman [27] described in the “*Modularity*” section.

### Data

In the Results section, we first analyze the performance of the community detection algorithms with artificially generated benchmark networks, and then we study protein interaction networks of different species. A description of these networks is provided in the following subsections.

#### Benchmark networks

The benchmark networks are generated by an algorithm proposed by [34]. It has been introduced with the purpose to generate benchmark networks for testing module detection algorithms. The generation of the network proceeds along the following steps.

1. The degree, *d*, of each node is randomly assigned from the power law distribution with exponent *γ*, in our case it is 1. The degree distribution is assigned depending on the maximum degree *d*_*max*_={20,40} and the average degree, *d*_*avg*_=10, selected as an input.
2. Nodes are assigned a fraction of edges, *μ*, that are shared with nodes of other communities and the remaining fraction, 1−*μ*, is shared within the community.
3. A community-size *k*_*min*_ and *k*_*max*_ is assigned in a following way, where *k*_*min*_>*d*_*min*_ and *k*_*max*_>*d*_*max*_ so that each node can be assigned to a community. The community size is decided based on the power law distribution so that the sum of the nodes in all communities is equal to the number on nodes in the network.
4. First, nodes are not assigned to any community and than nodes are assigned randomly to a community if the community-size exceeds the number of neighbours of the node in the community. This step is repeated until all nodes are assigned to a community.
5. In order to ensure that each node has a right approximation of *μ* and 1−*μ* for external and internal edges several rewiring steps are iterated.

For our analysis, we generated networks of vertex-size, |*V*|=1000, by varying different parameters for non-overlapping communities which are average degree, maximum degree, minimum cluster size, maximum cluster size and mixing parameter *μ*= {0.05, 0.10, 0.15, 0.20, 0.25, 0.30, 0.40, 0.50}.

#### Protein interaction networks

The protein interaction networks (PINs) we use for our analysis are obtained from *Biogrid database* [35]. In total, we use 10 PINs from 10 different species. The details are described in Table 1. These networks are pre-processed using the R package *igraph* [36] by extracting the giant connected components (GCC) of the networks.

**Table 1:** A list of protein networks used for detecting communities by different community detection algorithms

Tax id	Biological	No. of	No. of	Edge
	Name	vertices	interactions	density
10090	House mouse	5057	11560	0.000904
10116	Norway rat	1710	2582	0.001767
237561	Candida albicans SC5314	304	316	0.006860
284812	Schizosaccharomyces pombe 972h	3854	55054	0.007414
36329	Plasmodium falciparum 3D7	1172	2415	0.003519
3702	Arabidopsis Thaliana	7103	17752	0.000703
559292	Saccharomyces cerevisiae S288c	6008	227836	0.012620
6239	Caenorhabditis elegans	3701	7695	0.001123
7227	Drosophila melanogaster (fruit fly)	8017	38973	0.001212
9606	Homo sapiens	15795	159278	0.001276

As one can see in Table 1 these biological networks show a large variety in the network parameters such as number of nodes and number of edges.

### Normalized mutual information (NMI)

In order to assess the predicted modules of the algorithms qualitatively, we use the normalized mutual information (NMI) [37–39].

The normalized mutual information is defined as follows. Suppose we have two community detection algorithms, *U* and *V* and they predict |*R*| and |*C*| communities in a network. The overlap between the two predicted communities is shown in the contingency Table 2, i.e., community *U*_2_ and *V*_1_ share *n*_21_ nodes. Then the NMI [37–39] is calculated as follows.

$$NMI_{max} = \frac{I(U, V)}{H(U) + H(V)} $$

where

$$\begin{aligned} H(U)&= -\Sigma_{i=1}^{R} \frac{a_{i}}{N} \left(log\frac{a_{i}}{N}\right)\\ H(V)&= -\Sigma_{i=1}^{C} \frac{b_{i}}{N} \left(log\frac{b_{i}}{N}\right)\\ I(U,V)&=\Sigma_{i=1}^{R} \Sigma_{j=1}^{C}\frac{n_{ij}}{N} \left(log\frac{n_{ij}/N}{a_{i} b_{j}/N^{2}}\right) \end{aligned} $$

**Table 2:** A contingency table which defines overlap between two communities, *U* and *V*

U *↓*∖ V →	*V*_1_	*V*_2_	.	.	.	*V*_*C*_	Sums
*U*_1_	*n*_11_	*n*_12_	.	.	.	*n*_1*C*_	*a*_1_
*U*_2_	*n*_21_	*n*_22_	.	.	.	*n*_2*C*_	*a*_2_
.	.	.	.	.	.	.	.
.	.	.	.	.	.	.	.
*U*_*R*_	*n*_*R*1_	*n*_*R*2_	.	.	.	*n*_*RC*_	*a*_*R*_
Sums	*b*_1_	*b*_2_	.	.	.	*b*_*C*_	*N*

## Results

### Benchmark networks

We start our analysis investigating the performance of community detection algorithms by application to benchmark networks. The benchmark networks are generated by an algorithm [34], as described in the Methods section, that result in networks with a predefined modularity structure. Hence, it is know that the networks have a module structure and can be used as a reference to quantify the performance of the community detection algorithms in an objective manner.

In the following, we study various parameters of the benchmark algorithm to generate benchmark networks. Specifically, we set the network size to |*V*|=1000 nodes, for the average degree of the vertices we use $d_{i}^{avg} = 10 $ and for the maximum degree, $d_{i}^{max} = 20$. The minimum community-size parameter, we vary for *k*_*min*_={10,20,50,70,100,150} and the maximum community-size parameter for *k*_*max*_={20,50,70,100,150,200}. For the mixing parameter, we study values in the set *μ*={0.05,0.10,0.15,0.20,0.25,0.30,0.40,0.50}. For each parameter combination, we generate 50 networks, resulting in a population of benchmark networks with the same characteristics but random variations. This allows an assessment of the robustness of the results due to stochastically ocuring structural changes in the networks.

**Fig. 1:**
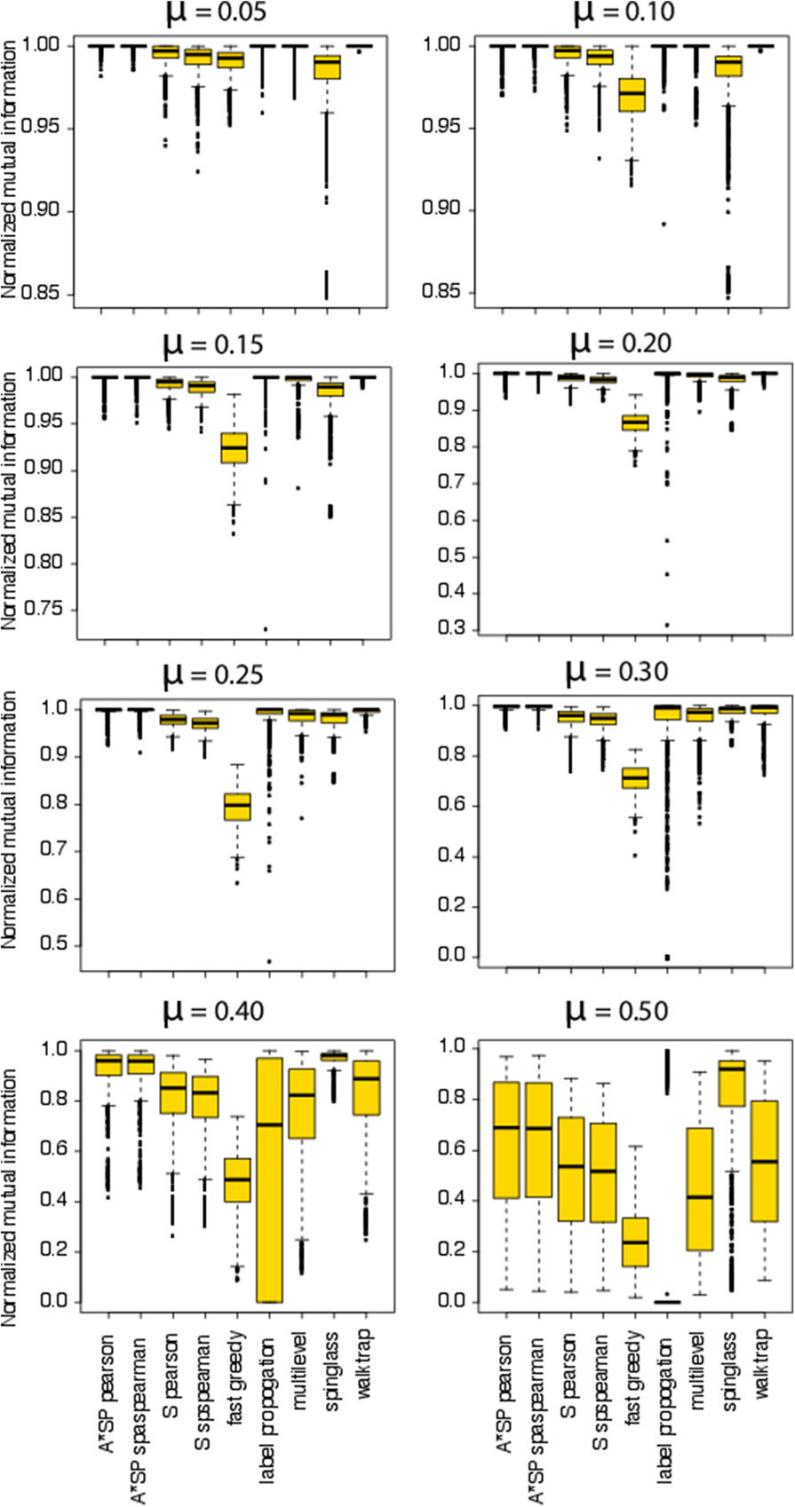
Normalized mutual information of different module detection algorithms for the benchmark networks

As performance measure for assessing the predicted modules of the community detection algorithms we are using the normalized-mutual information (NMI); see Methods section. The NMI evaluates the comparison of the true communities and the predicted communities, as identified by the different algorithms. The distribution of NMI values for different community detection algorithms is shown in Fig. 1. The parameters studied are: (a) Mixing parameter *μ*=0.05, average number of modules is 33 (b) Mixing parameter *μ*=0.1, average number of modules is 20 (c) Mixing parameter *μ*=0.15, average number of modules is20 (d) Mixing parameter *μ*=0.2, average number of modules is 20 (e) Mixing parameter *μ*=0.25, average number of modules is 21 (f) Mixing parameter *μ*=0.3, average number of modules is 33.

Overall, the figure shows that as the mixing parameter, *μ*, increases the performance of all module detection algorithms deteriorates. Compared to all algorithms, the Label propagation algorithm underperforms throughout all values of *μ* and the Spinglass community algorithm performs better than all other algorithms, except for low values of the mixing parameter. This indicates that the method has an optimal working point for intermediately connected modules, which is a counterintuitive behavior. Furthermore, our distance measure-based approaches, notably A*SP Pearson and A*SP Spearman, are showing in general a good performances, and compared to Fast greedy and Walktrap they show even a favourable performance.

### Performance of module detection algorithms by adding random edges

**Fig. 2:**
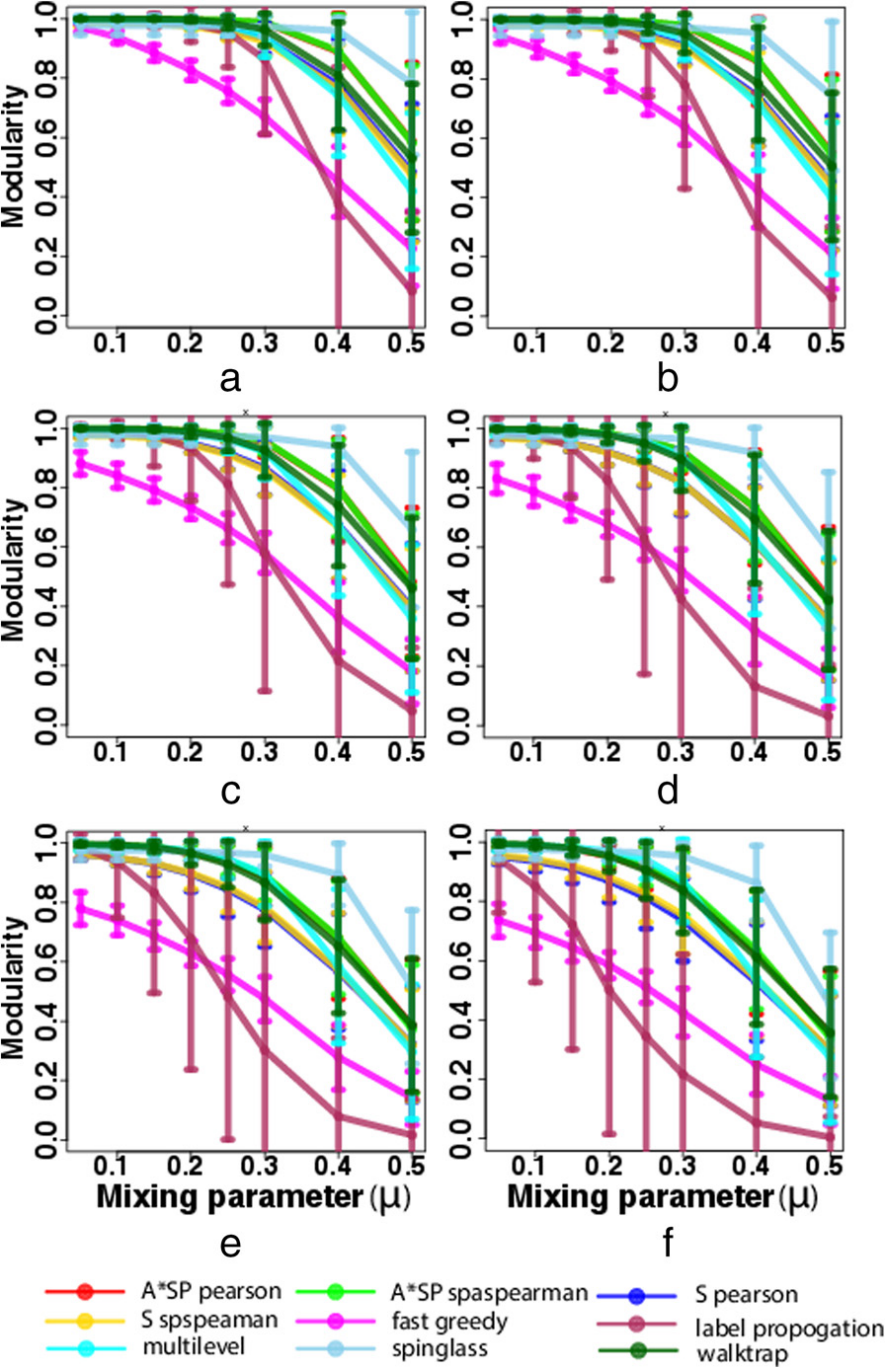
A comparison of modularity of different module detection algorithms by showing plots between modularity and mixing parameter (*μ*) in synthetic networks. The synthetic networks are modelled by adding certain percentage of random edges in the networks. **a** 5 *%* (**b**) 10 *%* (**c**) 20 *%* (**d**) 30 *%* (**e**) 40 *%* (**f**) 50 *%* additional edges of total edges are randomly added in synthetic networks

In this analysis, we test the robustness of different module detection algorithms against noise by adding a certain percentage of edges randomly to the network. Specifically, in the first step we generate synthetic networks, *G*=(*V*,*E*), with *N* modules as described in section *Benchmark networks*. Then we add a certain fraction of random edges resulting in *G*^′^=(*V*,*E*^′^), where, *E*^′^=*E*∪*E*^′′^ with *E*^′′^ is a randomly chosen set of edges between vertices in *V* of the benchmark network *G*. We then compare the modularity of the modules predicted by different module detection algorithms in *G*^′^ with the modules in *G*. The main objective of this analysis is to test the robustness of the module detection algorithms with respect to the addition of random edges to the network. The results of the performance of different module detection algorithms are shown in Fig. 2. In this figure we generated plots between modularity and mixing parameter (*μ*). From this analysis we find that the modularity of the modules predicted by different algorithms decrease as the percentage of added edges increases. The decrease in modularity is larger when the mixing parameter is higher. However, a small fraction of added edges do not effect the modularity, which can be seen in Fig. 2a and b. From this analysis we find that the fast greedy and label- propagation algorithms are the worse performing algorithms, for higher values of the mixing parameter (*μ*) the label propagation performs worse and for lower mixing parameter (*μ*) the fast greedy performs worse compare to other algorithms. The spinglass algorithm performs best in all cases, the multi-level algorithm also performs better but for higher mixing parameter (*μ*) the walktrap community and the clustering algorithms show a slightly better performance than the multi-level algorithm.

### Biological networks

Next, we extend our investigation to biological networks. Specifically, we use 10 PPI networks from different species. Details of these networks can be found in Table 1.

#### Modularity in PPI networks

First, we estimate the modularity, *Q*, and the number of modules in these PPI networks for the 9 community detection algorithms. The results of this analysis are shown in Tables 3 and 4 respectively.

**Table 3:** Modularity, *Q*, of PPI networks detected by different module detection algorithms

Tax id	$D_{M_{pearson}}\phantom {\dot {i}\!}$	$D_{M_{spearman}}\phantom {\dot {i}\!}$	$D_{{sp}_{pearson}}\phantom {\dot {i}\!}$	$D_{sp_{spearman}}\phantom {\dot {i}\!}$	Fast greedy	Label propogation	Multilevel	Spinglass	Walktrap
House mouse	0.4903	0.4168	0.4281	0.3999	0.5647	0.4578	0.6066	**0.6239**	0.5265
Norway rat	0.5061	0.2901	0.4985	0.4948	0.6608	0.5089	0.6682	**0.6683**	0.5951
Candida albicans SC531	0.4571	0.4629	0.4625	0.4629	0.4757	0.428	0.4757	0.4728	0.4689
Schizosaccharomyces pombe 972h	0.1669	0.1673	0.1005	0.128	0.2396	3e-04	0.2516	**0.268**	0.1545
Plasmodium falciparum 3D7	0.4775	0.4576	0.4713	0.466	0.5171	0.0066	0.5222	**0.5396**	0.3505
Arabidopsis Thaliana	0.6635	0.6004	0.5824	0.5781	0.6893	0.6977	0.7296	**0.742**	0.6822
Saccharomyces cerevisiae S288c	0.2108	0.2055	0.0399	0.0283	0.2557	1e-04	0.2532	0.2741	0.2221
Caenorhabditis elegans	0.5141	0.5087	0.5023	0.4989	0.6042	0.1872	0.6106	**0.6231**	0.5268
Drosophila melanogaster	0.4509	0.4491	0.4124	0.4238	0.471	0.2608	0.5232	**0.5307**	0.3865
Homo sapiens	0.2045	0.0898	0.0708	0.0655	0.2877	1e-04	0.3498	**0.3612**	0.253
Average modularity	0.4141	0.3648	0.3568	0.3546	0.4765	0.2547	0.4990	0.5103	0.4166

The bold values show two highest modularities of the modules predicted by module detection algorithms

**Table 4:** Number of modules of PPI networks detected by different module detection algorithms

Tax id	$D_{M_{pearson}}\phantom {\dot {i}\!}$	$D_{M_{spearman}}\phantom {\dot {i}\!}$	$D_{{sp}_{pearson}}\phantom {\dot {i}\!}$	$D_{sp_{spearman}}\phantom {\dot {i}\!}$	Fast greedy	Label propogation	Multilevel	Spinglass	Walktrap
House mouse	9	8	10	6	71	95	28	25	360
Norway rat	26	4	42	55	28	56	28	25	123
Candida albicans SC531	16	14	12	14	14	11	14	12	13
Schizosaccharomyces pombe 972h	5	11	2	2	20	5	8	13	582
Plasmodium falciparum 3D7	15	16	25	26	18	4	22	22	179
Arabidopsis Thaliana	15	13	20	14	57	190	36	25	390
Saccharomyces cerevisiae S288c	14	10	8	13	5	2	8	10	319
Caenorhabditis elegans	10	6	9	6	38	51	29	25	351
Drosophila melanogaster	21	21	20	19	55	24	29	25	884
Homo sapiens	30	20	34	63	89	3	13	21	3425

The first observation we make is that the best performing algorithms are the Multilevel and the Spinglass community algorithms. Interestingly, for some organisms, e.g., *Schizosaccharomyces pombe* and *Homo sapiens*, the Label propagation algorithm almost fails entirely to detect communities. In contrast, Fast-greedy and Walktrap are also finding acceptable modularity values for the networks for which the Label propagation algorithm has problems. Among the distance-based measures, $D_{M_{pearson}}\phantom {\dot {i}\!}$, is the best performing method.

For the predicted number of modules, the Walktrap algorithm results in many more modules than any other method, whereas the remaining methods predict a comparable number of modules. For instance, for the PPI network of *Homo Sapiens* (9606), Walktrap predicts 38 times more modules than Fast-greedy and 163 times more modules than the Spinglas method. This is interesting because this is not beneficially reflected in the modularity values *Q*, see Table 3, in a way that this would lead to superior modularity values.

**Fig. 3:**
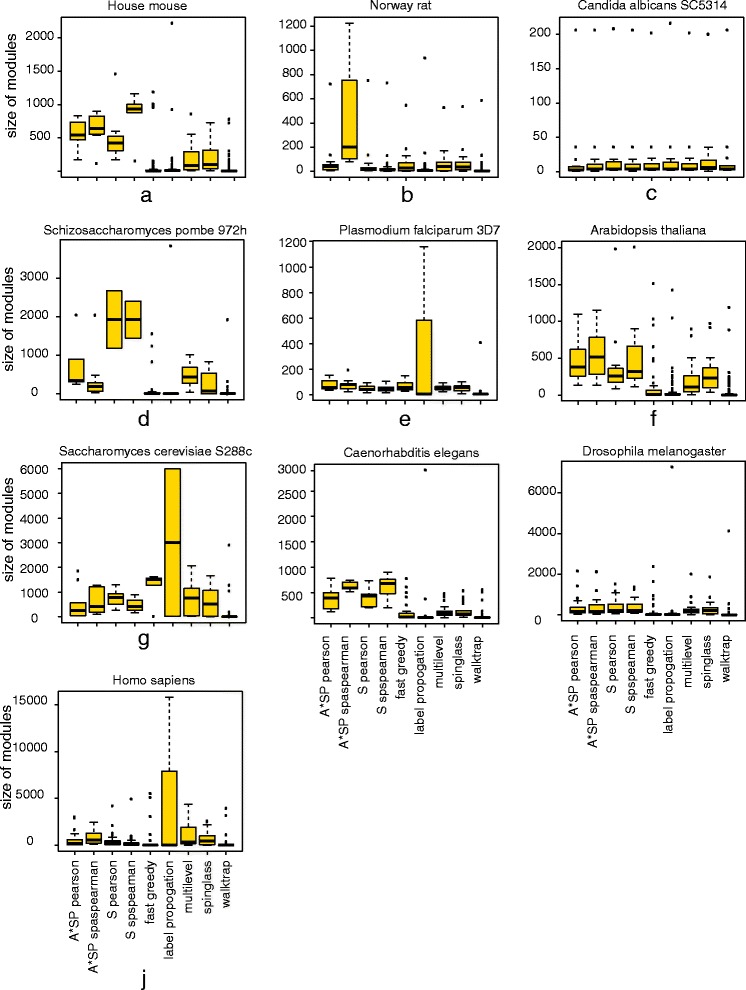
Distribution of the size of modules detected in PPI networks by different module detection algorithms

Aside from the number of predicted modules, it is important to know the size distribution of these, i.e., how many proteins belong to the corresponding modules. The distributions of the sizes of the modules for the studied organisms are shown in Fig. 3. Here one can see that there is a considerable variation among the methods. For instance, the variation of module sizes predicted by Walktrap are generally smaller for all organisms. This is understandable because the predicted number of modules is for this method by far the largest, which leads in general to rather small modules. In contrast, the variations for the correlation-based methods depend crucially on the organism. Overall, the largest variability is observed for the Label propagation algorithm.

Considering the agreement among different methods, the module structure of *Candida albicans* is least different and, hence, shows the highest level of consensus. For this organism, even Walktrap results in a moderate number of predicted modules, which is comparable to all other methods.

**Fig. 4:**
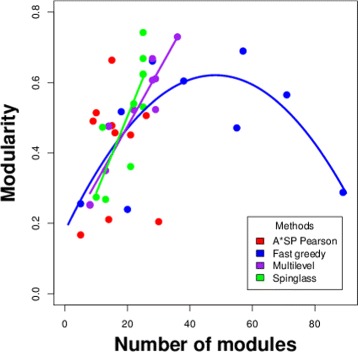
Scatter plot between the number of modules and the modularity. Each method is color coded by a different color. The shown curves correspond to Least Squares regression models. For A*SP Pearson, no statistically significant model could be fit that would be different from a horizontal line

In Fig. 4 we combine the results from Tables 3 and 4 as a scatter plot between the number of modules and the modularity. For reasons of clarity, we show only results for four out of the nine methods because the other algorithms add nothing for the following discussion. The interesting observation is that Fast greedy displays a curious behavior because for an increasing number of predicted modules in the networks, the modularity decreases. In order to quantitatively confirm this observation we fit a polynomial regression of second order by the Least Squares method minimizing the residual sum of squares (RSS). For the linear and the quadratic term we obtain *p*-values of 0.0194 and 0.0211, which are significant for *α*=0.05. This confirms our observation statistically. In contrast, Multilevel and Spinglass can be approximated by a linear regression model, with *p*-values of 10^−5^ and 0.004.

Interestingly, the A*SP Pearson algorithm is somehow located between these models in the sense that the best linear fit would only use an intercept but no slope and the quadratic regression is barely not significant with *p*-values of 0.08 for both the linear and quadratic term but higher values of adjusted *R*^2^ values. For this reason, we do not include results form the regression in Fig. 4.

#### Comparison of algorithms

In order to investigate the similarity of the identified modules for different algorithms in detail, we use again the NMI measure. However, this time we use the NMI to compare the predicted community structure of one method with the predicted community structure of another method. In this way, the similarity of the predicted communities is assessed. In other words, this analysis will provide us with information about the consistency of results among different methods but does not allow to gain insights into the absolute quality of the predicted module structures, because the ground truth does not enter this analysis.

**Fig. 5:**
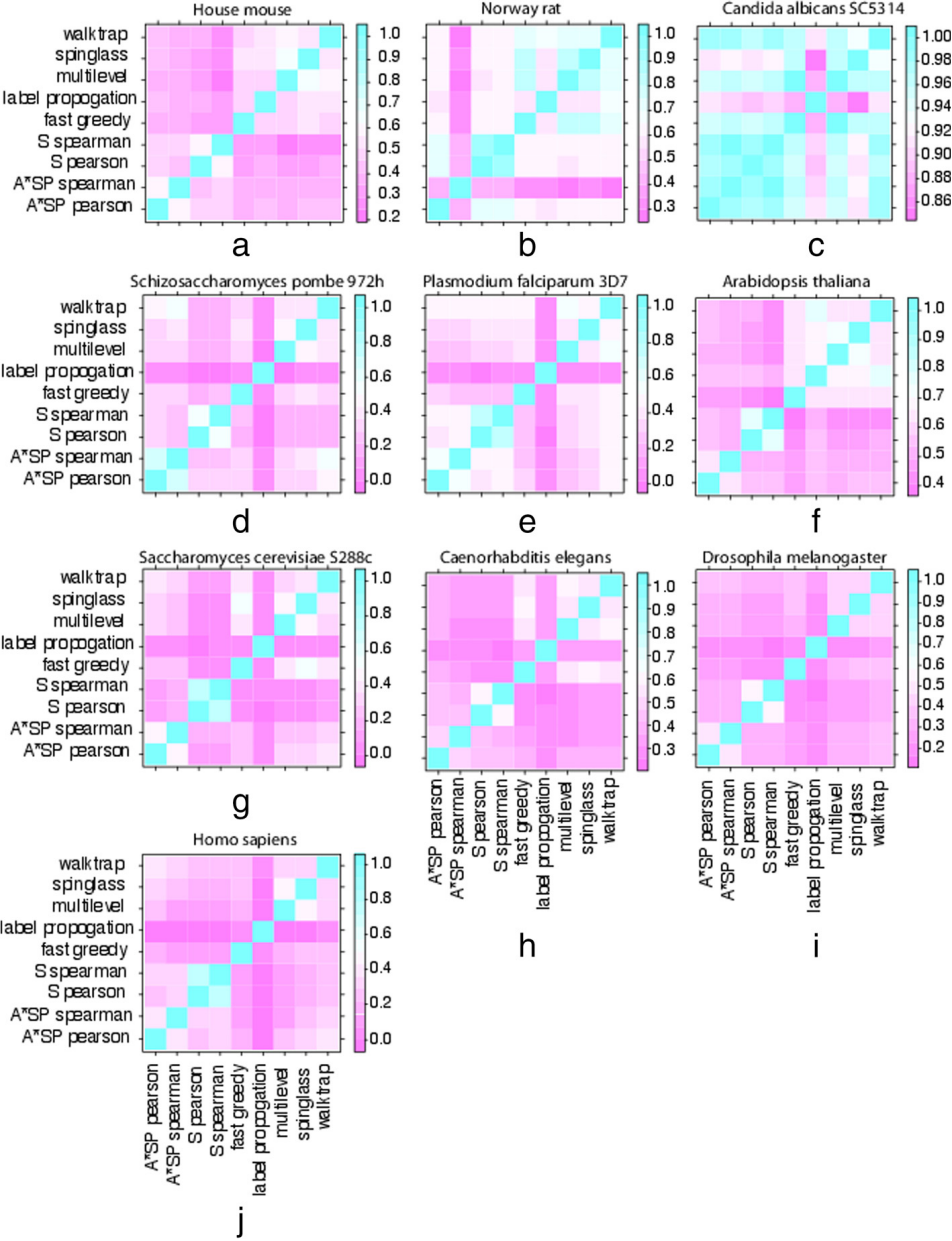
Similarity of the predicted module structures in PPI networks assessed by the NMI. The values of the NMI are color coded, as indicated by the color bar in each figure, showing the range of assumed values

The results of this analysis are shown in the form of level plots of the NMI values between different community detection algorithms in Fig. 5. The color code of the NMI values goes from violet (low values) to blue (high values), see Fig. 5 for the different scales for the different organisms. In general, there is a good agreement among different methods, however, on a moderate level. For instance, for *Drosophila melanogaster* the NMI values are around ∼0.4. Similarly, for House mouse and *Homo sapiens*. In contrast, for Norway rat the NMI values for A*SP Spearman are succinctly lower than from all other algorithms. Also Label propagation stands out in a similar way for *Plasmodium falciparum* and yeast.

By looking at the scale of the NMI values, one can see that for *Candida albicans* the lower values of the scale assumes higher values than for all other organisms, ranging from 0.86 to 1.00. This indicates that the similarity among all community detection algorithms is for this PPI networks highest, confirming our observation in Fig. 3, where we have seen that the variation of the size of modules is for all methods similar and quite small. Finally, we want to note that, in general, the distance-based measures are showing a higher similarity among each other than to the other community detection algorithms.

#### Robustness of module detection regarding perturbations

Our next analysis investigates the robustness of the predicted modules for perturbed PPI networks. Specifically, we test how a module detection algorithm changes its performance if some interactions in a PPI network are randomly deleted. The rationale of our analysis is based on the assumption that biological networks, and the interactions they are made of, are not known with absolute certainty. Instead, some interactions present in our PPI networks may be false positives due to measurement errors. Since all PPI networks we are using are inferred from experimental data, we think this assumption is very reasonable.

In order to study the effect of false positive interactions, we generate 20 perturbed networks for each PPI network, $G^{sub}_{1}, G^{sub}_{2} \dots G^{sub}_{20}$, by deleting randomly 5 *%* of the edges in a PPI network. In order to make sure the the resulting networks are still connected, we remove only edges from nodes having a degree of *D*(*v*_*i*_,*G*)≥2 and prevent removal of the last remaining edge. Then, we apply the community detection algorithms to the networks, $G^{sub}_{1}, G^{sub}_{2} \dots G^{sub}_{20}$, and compare the predicted modules with the results from the unperturbed PPI network by using the NMI.

**Fig. 6:**
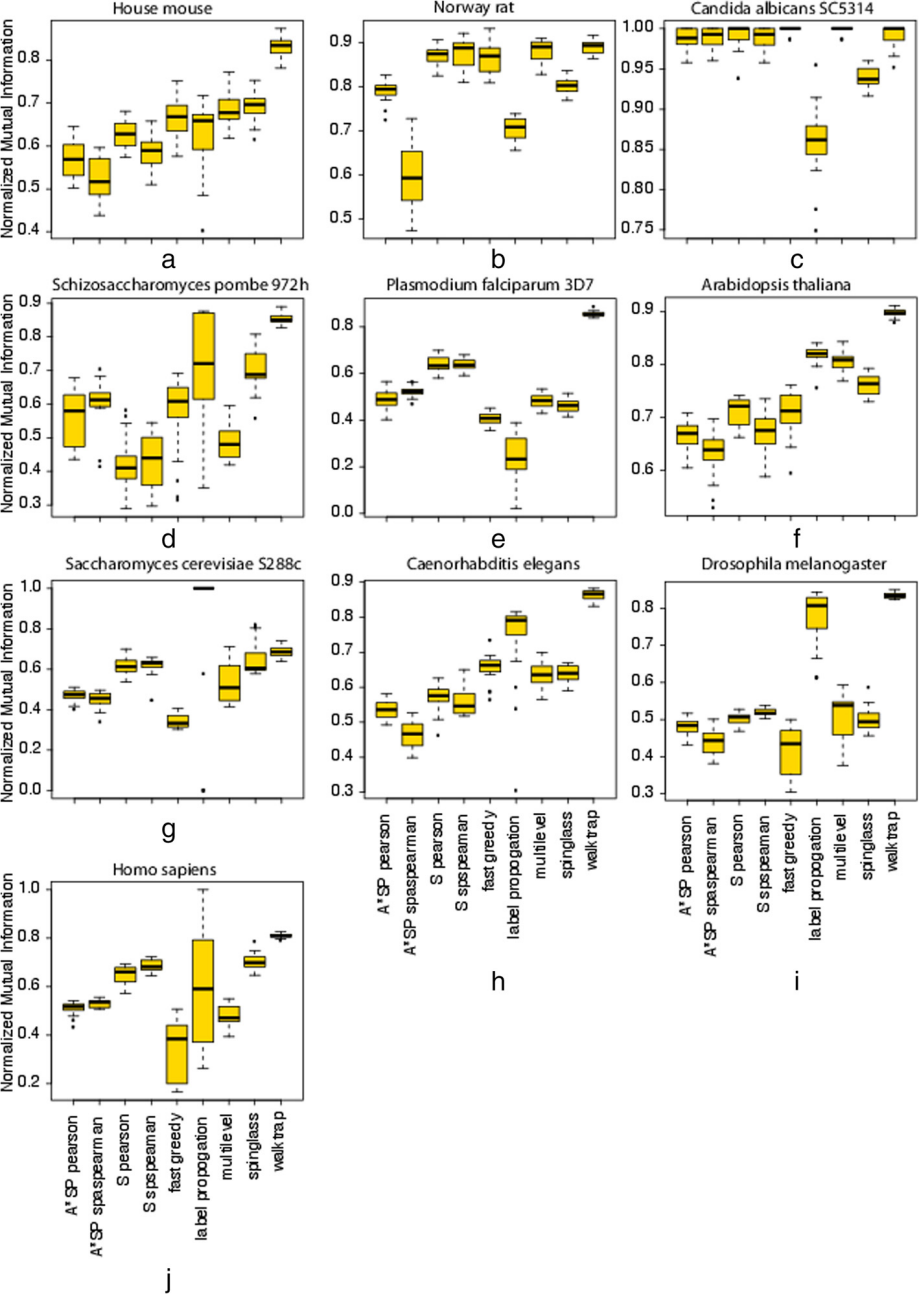
Robustness of module detection regarding perturbation of the PPI networks. Distribution of NMI values comparing communities obtained from the unperturbed and perturbed PPI networks generated by randomly deleting 5 *%* of the edges

The results of this analysis are shown in Fig. 6. The first observation we make is that in all but two cases the NMI values are considerably smaller than 1.00, indicating a large change in the predicted communities. One exception is the Label propagation algorithm for *Saccharomyces cerevisiae* and the other is for all algorithms but Label propagation and Spinglass for *Candida albicans*. For all other algorithms and the remaining organisms, the obtained NMI values are much smaller, with the lowest value observed for Label propagation for *Plasmodium falciparum*. In general, compared to other methods and across the organisms, the most robust method appears to be Walktrap.

Overall, the results show that even a moderate change in a PPI network leads, usually, in quite large changes of the predicted module structure, regardless of the algorithm or the organism.

#### Biological meaning of predicted modules

As far, we focused on more technical aspects of predicted modules. Now we switch gears by investigating the biological meaning of these modules. We do this by using external information, not included in the network structure itself, for assessing the predicted modules. As source for this external information we are using the Gene Ontology (GO) database [32] that provides comprehensive information about the involvement of genes across many organisms in diverse biological processes.

Specifically, we performed an enrichment analysis of biological pathways obtained from the GO database for the modules detected by the community detection algorithms. In order to test the statistical significance of biological pathways, corresponding to an over-representation of genes from a particular biological process, we use Fisher’s exact test. Since we are conducting 1000s of hypothesis tests, we need to apply a multiple testing correction. For this reason, we apply a conservative Bonferroni correction for a significance level of 0.001. The results of this analysis are shown in Table 5.

**Table 5:** Number of statistically significant pathways as identified by a Fisher’s exact test that are enriched in the predicted modules in the PPI networks

Tax id	$D_{M_{pearson}}\phantom {\dot {i}\!}$	$D_{M_{spearman}}\phantom {\dot {i}\!}$	$D_{{sp}_{pearson}}\phantom {\dot {i}\!}$	$D_{sp_{spearman}}\phantom {\dot {i}\!}$	Fast greedy	Label propogation	Multilevel	Spinglass	Walktrap	Total pathways
House Mouse	608	617	476	477	**949**	818	817	801	**903**	7057
Norway rat	182	35	159	164	265	97	**315**	**311**	147	5012
Schizosaccharomyces	33	48	11	8	25	2	66	**78**	**98**	1115
pombe 972h										
Plasmodium falciparum 3D7	0	0	0	0	0	0	0	0	0	51
Arabidopsis Thaliana	466	458	485	433	657	**834**	747	668	**869**	2506
Saccharomyces cerevisiae	780	772	265	199	944	10	**1019**	**993**	883	3236
S288c										
Caenorhabditis elegans	60	114	104	105	**203**	101	145	**203**	**249**	1823
Drosophila melanogaster	757	778	618	640	700	206	812	**837**	**863**	3984
Homo sapiens	1277	762	467	536	1321	8	**1863**	2011	1824	9371

The bold values show the respective number modules predicted by them

In the last column of this table, the total number of tested biological processes is shown as a reference. Overall, the Multilevel and Spinglass community detection algorithms have the largest number of enrichment biological pathways. But in general, these numbers are not too far apart from the remaining methods, with some exceptions. It is interesting to note that for *Plasmodium falciparum* (36,329) none of the algorithms predicts modules that contain at least one enriched pathway. The reason for this may be in the very small number of total pathways (51) tested for this organism.

In Table 6, we show the the percentage of enriched pathways. The highest percentage is observed for *Arabidopsis Thaliana* (3702), *Saccharomyces cerevisiae* (559,292) and *drosophila milanogaster* (7227) for different module detection algorithms. In contrast, Norway rat (10,116) leads to the least percentage ∼6 *%*.

**Table 6:** Percentage of statistically significant pathways (%) as identified by a Fisher’s exact test that are enriched in the identified modules in the PPI networks

Tax id	$D_{M_{pearson}}\phantom {\dot {i}\!}$	$D_{M_{spearman}}\phantom {\dot {i}\!}$	$D_{{sp}_{pearson}}\phantom {\dot {i}\!}$	$D_{sp_{spearman}}\phantom {\dot {i}\!}$	Fast greedy	Label propogation	Multilevel	Spinglass	Walktrap
House mouse	8.62	8.74	6.75	6.76	13.45	11.59	11.58	11.35	12.80
Norway rat	3.63	0.70	3.17	3.27	5.29	1.94	6.28	6.21	2.93
Schizosaccharomyces pombe 972h	2.96	4.30	0.99	0.72	2.24	0.18	5.92	7.00	8.79
Plasmodium falciparum 3D7	0.00	0.00	0.00	0.00	0.00	0.00	0.00	0.00	0.00
Arabidopsis Thaliana	18.60	18.28	19.35	17.28	26.22	33.28	29.81	26.66	34.68
Saccharomyces cerevisiae S288c	24.10	23.86	8.19	6.15	29.17	0.31	31.49	30.69	27.29
Caenorhabditis elegans	3.29	6.25	5.70	5.76	11.14	5.54	7.95	11.14	13.66
Drosophila melanogaster (fruit fly)	19.00	19.53	15.51	16.06	17.57	5.17	20.38	21.01	21.66
Homo sapiens	13.63	8.13	4.98	5.72	14.10	0.09	19.88	21.46	19.46

**Fig. 7:**
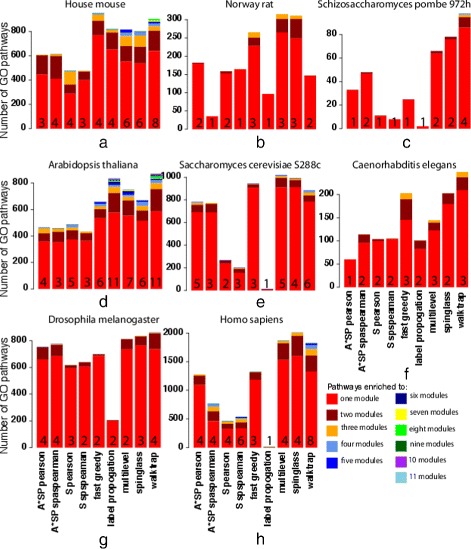
Bar plots of the number of pathways that are enriched in multiple modules. The numbers inside each bar correspond to the maximum number of modules to which pathways are enriched

The results in Tables 5 and 6 provide us with an overview of the enriched pathways, but they do not tell us if a pathway is enriched in just one predicted module or in several. This information is shown in Fig. 7. In this figure, we color-coded the number of pathways showing enrichment for multiple modules, ranging from 1 to 11 modules. The maximum number of modules is also shown as a number in the barplots, for each algorithm. From the shown results, we see that most pathways are only enriched in one module (red) indicating a biological specification of these modules. In general, the number of enriched pathways decreases with an increasing number of modules for all methods and across all organisms. These observations support the hypothesis that modules are used as functional units to carry out *specific* biological functions. In general, the modules predicted by the Walktrap community algorithm have a larger number of enriched pathways to multiple modules. Furthermore, the pathways of House mouse and *Arabidopsis thaliana* have a higher maximum number of pathways that are enriched for the maximum number of modules. The Label propagation algorithm predicts the lowest number of pathways enriched to multiple modules, except for *Arabidopsis thaliana*, which is a potential indicator of a poor predictability of modules in PPI networks. Another interesting aspect to remark, is that the algorithms Multilevel and Spinglass, which predicted modules with the highest modularity, are having in general the largest number of enriched pathways to the maximum number of modules.

**Fig. 8:**
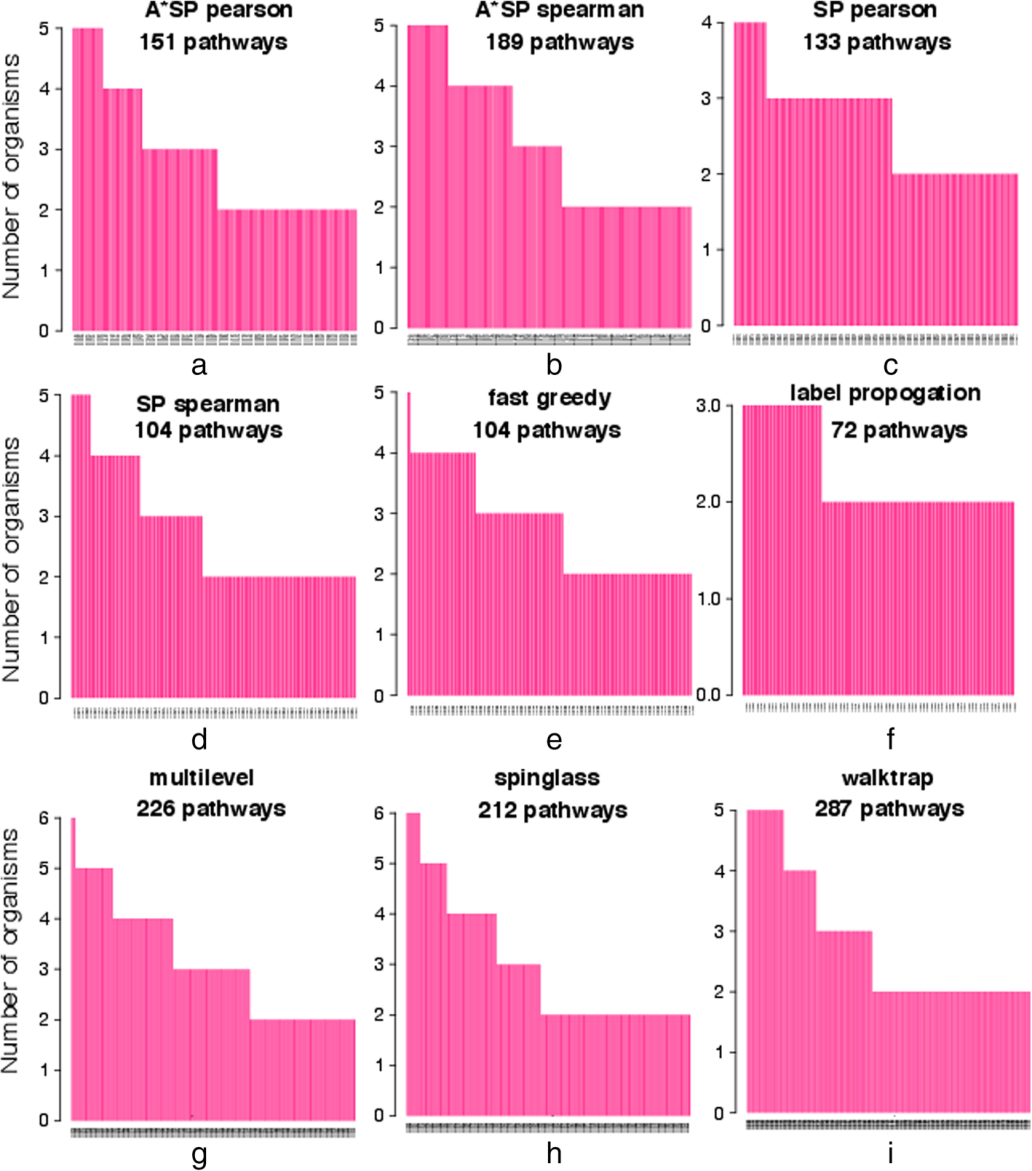
Bar plots of pathways which are enriched in two or more organisms. The numbers in each figure are showing the total number of pathways that are enriched

Next, we study the significant pathways that are common across different organisms. Specifically, in Fig. 8, we plot a distribution of common pathways. The Multilevel and Spinglasss have three and ten pathways respectively in common among 6 organisms; see Table 7. These processes are mostly involved in metabolic processes and cell communication. Other algorithms, except Label propogation, predict pathways common in four to five organisms, while Label propagation, has pathways that are common in only three organisms. Overall, the Walktrap community algorithm predicts the largest number of 287, pathways that are common in at least two modules.

**Table 7:** GO pathways which are enriched to more than one modules predicted by *spinglass* and *multilevel* community detectin algorithms that are common among 6 organisms (see Fig. 8)

Common GO pathways		
Algorithm	GO Pathways	Name
Multilevel	GO:0006139	Nucleobase-containing compound
		metabolic process
	GO:0007154	Cell communication
	GO:0090304	Nucleic acid metabolic process
Spinglass	GO:0006139	Nucleobase-containing compound
		metabolic process
	GO:0006725	Cellular aromatic compound metabolic process
	GO:0006807	Nitrogen compound metabolic process
	GO:0010467	Gene expression
	GO:0016070	RNA metabolic process
	GO:0034641	Cellular nitrogen compound metabolic process
	GO:0044260	Cellular macromolecule metabolic
		process
	GO:0046483	Heterocycle metabolic process
	GO:0090304	Nucleic acid metabolic process
	GO:1901360	Organic cyclic compound metabolic process

### Subnetwork analysis of Homo sapiens obtained from different experimental methods

**Fig. 9:**
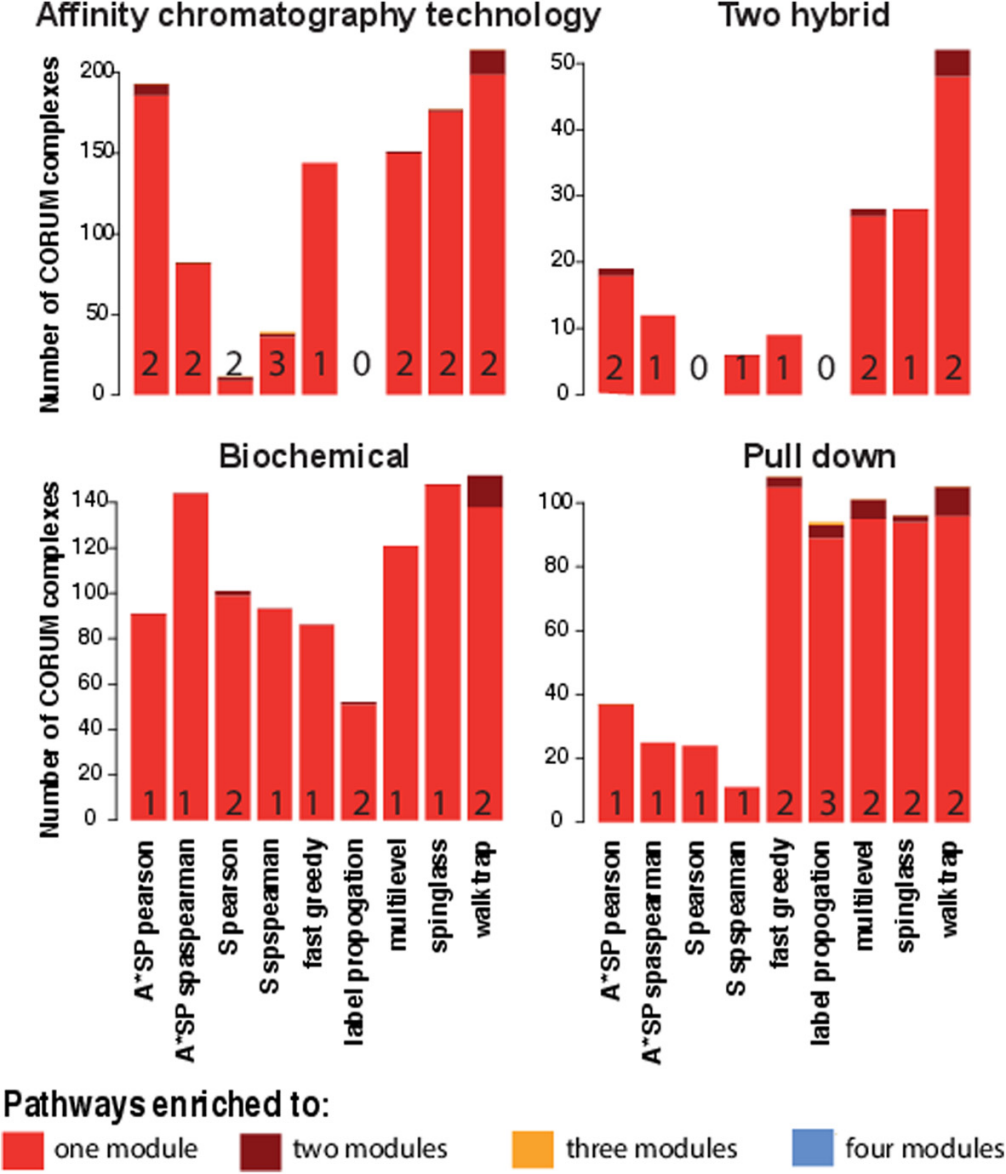
Bar plots of the number of pathways (CORUM complex) that are enriched in multiple modules of PPI subnetworks of *Homo Sapiens* from different experimental types. The numbers inside each bar correspond to the maximum number of modules to which pathways are enriched

We extend our investigation to the subnetworks of *Homo sapiens*. Specifically, we use the 4 largest connected PPI sub-networks from different experimental methods. Details of these networks can be found in Table 8. We estimate the modularity, *Q*, and the number of modules in these PPI networks for the 9 community detection algorithms. The results of our analysis are shown in Tables 9 and 10 respectively. The modularity of the subnetwork obtained from *Affinity chromatography technology* showing a slightly higher modularity for fastgreedy, multilevel and spinglass algorithms. However, for other subnetworks the modularity is considerably higher compared to the complete PPI network of *Homo Sapiens*. The modularities of of subnetworks highlight the fact that different subnetowrks obtained from different experimental methods provide a mixture of different structural properties of the complete PPI network. The analysis also highlights the fact that *multilevel* and *spinglass* algorithms are consistently performing better than other algorithms and *walktrap* community predicts more number of modules compare to other algorithms. Also the clustering based algotrithms and label propogation algorithms which perform better in synthetic networks are showing lowest modularity. In the next step of the analysis we performed enrichment analysis of pathways obtained form the CORUM complex database [40]. The results of this analysis are shown in Tables 11 and 12. The percentage of enriched pathways of CORUM complex database are higher compare to the GO pathways for individual organisms except the subnetwork obtained from *two hybrid* experimental data. In the next step we predicted that if a pathway is enriched in just one predicted module or in several. This information is shown in Fig. 9. In this figure, the color-coded barplots show the number of pathways showing enrichment for multiple modules, ranging from 1 to 4 modules. In this analysis a large fraction of pathways are enriched to just one modules and a few pathways are enriched to two or three modules predicted by different module detection algorithms. A list of pathways which are enriched to more than one modules predicted by multilevel and spinglass algorithms are shown in Table 13.

**Table 8:** Subnetwork of PPI interactions of Human obtained from different experimental types

Experiment type	No. of vertices	No. of interactions	Edge density
Affinity chromatography	13124	82900	0.000962
Two hybrid	9844	37280	0.000769
Biochemical	3686	20083	0.00295
Pull down	5714	10957	0.00067

**Table 9:** Modularity, *Q*, of PPI subnetworks detected by different module detection algorithms

Experimental type	$D_{M_{pearson}}\phantom {\dot {i}\!}$	$D_{M_{spearman}}\phantom {\dot {i}\!}$	$D_{{sp}_{pearson}}\phantom {\dot {i}\!}$	$D_{sp_{spearman}}\phantom {\dot {i}\!}$	Fast greedy	Label propogation	Multilevel	Spinglass	Walktrap
Affinity chromatography	0.057	0.027	0.079	0.078	0.319	0.0004	**0.352**	**0.372**	0.221
Two hybrid	0.424	0.418	0.371	0.368	0.456	0.0030	**0.495**	**0.508**	0.406
Biochemical	0.572	0.575	0.515	0.527	0.529	0.0715	**0.585**	**0.611**	0.512
Pull down	0.535	0.424	0.450	0.446	0.649	0.5650	**0.666**	**0.676**	0.569

The bold values in show two highest modularities of the modules predicted by module detection algorithms

**Table 10:** Total number of modules of PPI subnetworks detected by different module detection algorithms

Experimental type	$D_{M_{pearson}}\phantom {\dot {i}\!}$	$D_{M_{spearman}}\phantom {\dot {i}\!}$	$D_{{sp}_{pearson}}\phantom {\dot {i}\!}$	$D_{sp_{spearman}}\phantom {\dot {i}\!}$	Fast greedy	Label propogation	Multilevel	Spinglass	Walktrap
Affinity chromatography	104	12	11	42	70	7	11	23	4215
Two hybrid	19	14	18	20	88	15	27	25	995
Biochemical	n 9	15	13	10	58	31	24	22	604
Pull down	11	7	17	13	75	156	46	25	370

**Table 11:** Total number of significant CORUM complexes enriched to atleast one module of PPI subnetworks detected by different module detection algorithms

Experimental type	$D_{M_{pearson}}\phantom {\dot {i}\!}$	$D_{M_{spearman}}\phantom {\dot {i}\!}$	$D_{{sp}_{pearson}}\phantom {\dot {i}\!}$	$D_{sp_{spearman}}\phantom {\dot {i}\!}$	Fast greedy	Label propogation	Multilevel	Spinglass	Walktrap	Total pathways
Affinity chromatography	**193**	82	11	39	144	0	151	177	**214**	431
Two hybrid	19	12	0	6	9	0	**28**	**28**	**52**	361
Biochemical	91	144	101	93	86	52	121	**148**	**152**	325
Pull down	37	25	24	11	**108**	94	101	96	**105**	321

The bold values show the respective number modules predicted by them

**Table 12:** Total percentage of significant CORUM complexes enriched to atleast one module of PPI subnetworks detected by different module detection algorithms

Experimental type	$D_{M_{pearson}}\phantom {\dot {i}\!}$	$D_{M_{spearman}}\phantom {\dot {i}\!}$	$D_{{sp}_{pearson}}\phantom {\dot {i}\!}$	$D_{sp_{spearman}}\phantom {\dot {i}\!}$	Fast greedy	Label propogation	Multilevel	Spinglass	Walktrap
Affinity chromatography	0.448	0.190	0.026	0.090	0.334	0.000	0.350	0.411	0.497
Two hybrid	0.044	0.028	0.000	0.014	0.021	0.000	0.065	0.065	0.121
Biochemical	0.211	0.334	0.234	0.216	0.200	0.121	0.281	0.343	0.353
Pull down	0.086	0.058	0.056	0.026	0.251	0.218	0.234	0.223	0.244

**Table 13:** CORUM complexes which are enriched to more than one modules predicted by *spinglass* and *multilevel* community detectin algorithms

Common CORUM complexes	
Affinity chromatography technology	
Algorithm	Name
Multilevel	55S ribosome, mitochondrial
Spinglass	RNA polymerase II complex, chromatin structure modifying
Two hybrid	
Multilevel	C complex spliceosome
Spinglass	-
Biochemical	
Multilevel	-
Spinglass	-
Pull down	
Multilevel	PA700-20S-PA28 complex
	BRCA1-RNA polymerase II complex
	Spliceosome
	18S U11/U12 snRNP
	C complex spliceosome
	17S U2 snRNP
Spinglass	RNA polymerase II holoenzyme complex
	BRCA1-RNA polymerase II complex"

#### Time complexity of the algorithms

Finally, we show results for the time complexity of the community detection algorithms. In Table 14 the run time in seconds for the analysis of the PPI networks are shown. Overall, the fastest algorithm is Label propagation that provided for all studied networks the quickest results, below one second. For all other methods, even when they are in general fast, there is at least one network that requires much more time. For instance, Fast-greedy is in general quite fast and comparable to Label propagation, but for the networks *Saccharomyces cerevisiae* (559,292) and *Homo sapiens* (9606) it takes over 463 respectively 2287 times longer than for Label propagation. A similar observation can be made for Walktrap.

**Table 14:** Estimated time, in seconds, to detect modules in biological networks by different module detection algorithms

Tax id	$D_{M_{pearson}}\phantom {\dot {i}\!}$	$D_{M_{spearman}}\phantom {\dot {i}\!}$	$D_{{sp}_{pearson}}\phantom {\dot {i}\!}$	$D_{sp_{spearman}}\phantom {\dot {i}\!}$	Fast greedy	Label propogation	Multilevel	Spinglass	Walktrap
House mouse	231.8423	243.8301	230.2666	247.3037	1.1767	0.1042	0.0583	236.0281	1.6766
Norway rat	13.2490	12.9271	11.7737	13.0321	0.1114	0.0084	0.0102	45.1722	0.2000
Candida albicans SC5314	2.1909	0.1740	0.1604	0.1898	0.0091	0.0025	0.0019	6.5747	0.0354
Schizosaccharomyces pombe 972h	114.2011	116.9353	107.3772	116.8714	2.8521	0.0216	0.2264	468.3092	3.3914
Plasmodium falciparum 3D7	6.5812	5.2746	3.9287	4.3812	0.0227	0.0139	0.0242	43.3769	0.1493
Arabidopsis Thaliana	630.2055	650.8486	636.2166	651.3501	1.2628	0.0968	0.0748	346.2693	3.2913
Saccharomyces cerevisiae S288c	415.8757	430.9147	411.8197	422.0503	183.0446	0.0847	1.5457	2248.5317	115.8467
Caenorhabditis elegans	100.4278	101.1053	94.3029	100.3038	0.2025	0.0438	0.0318	119.1461	0.8575
Drosophila melanogaster (fruit fly)	887.6161	922.9284	889.0182	911.5044	5.5153	0.0796	0.2107	590.4792	7.1921
Homo sapiens	6750.1157	7134.5106	7056.7373	7336.6568	51.3895	0.1185	0.5939	2411.9311	48.2544
Average time	915.23	961.94	944.16	980.36	24.558	0.0574	0.2777	651.58	18.089

## Discussion and conclusion

In our analysis, we used 9 community detection algorithms to predict modules in PPI networks of 10 different organisms. Overall, our analysis provides a comprehensive understanding of the performance of large community detection algorithms. Also, our analysis highlights organism-specific differences of PPI networks and the biological meaning of the predicted modules.

Overall, from our analysis of these networks we found that the Spinglass, Multilevel and Fastgreedy algorithm preform in general much better than the other algorithms. Furthermore, the Multilevel and Fast greedy algorithm have, in addition, a good run time (see Table 14) that allows to obtain results for large networks within seconds. Interestingly, despite the fact that these three algorithms are performing better, there is no complete similarity among these algorithms in terms of the predicted modules, but the results are to a large extend method-specific. Another interesting fact about the Multilevel and Spinglass community algorithm is that the number of modules and the modularity are linearly correlated, while the performance of Fast greedy decreases as the number of modules increases (see Fig. 4). At this point it is unclear which behavior reflects the modularity vs number of modules dependency best for biological organisms. However, it appears reasonable to assume that there is a limiting factor in the growth of modularity of biological networks, which would suggest that the behavior of Fast greedy is a reflection of biological properties of the networks rather than a technical property or a bias of the method.

Although, we studied extensively the performance of modules in biological networks and found high modularity for some organisms, still, for some organisms, such as *Homo Sapiens* and *Saccharomyces cerevisiae*, we find a low modularity. This is especially surprising for *Homo Sapiens*. One reason for the low modularity in these networks could be the existence of many overlapping nodes between communities giving raise to overlapping modules and pathways. Therefore, the standard non-overlapping community prediction methods may not be optimally suitable for detecting communities in such organisms. This would suggest that more effort needs to be placed on the development of such algorithms, because only in this way one could shed light on the nature of the overlapping modular structure of PPI networks. Another explanation could be that the PPI networks contain incomplete information. One reason for this argument is because the highest modularity is predicted by the Spinglass algorithm for *Arabidopsis Thaliana* (3702), which is a less complex organism, and for this reason is easier to study. Also the modularity of *Arabidopsis Thaliana* (3702) is constantly predicted higher by all other algorithms.

By studying the biological meaning of predicted modules, we found that a large proportion of pathways is enriched in only a single module, in all organisms and for all algorithms. This underlines the role of biological pathways as part of a special functioning component in an organism. However, a small set of biological pathways is enriched in more than one module, and an even smaller proportion of pathways is commonly enriched to multiple modules in all organisms. In general the classification of these pathways can broadly be grouped into the following categories:

- Pathways which are part of a single module only across many organisms.
- Pathways which are part of multiple modules across many organisms.
- Pathways which are part of a single module and a single organisms.
- Pathways which are part of multiple modules and a single organisms.

It would be interesting to see what biological processes they contribute to and what role they play in different organisms in order to see changes in an evolutionary perspective or the emergence of a higher level of functioning in different organisms.

In summary, the identification of modules in networks is a very complex problem and more work needs to be done. A potential future direction could be to extend the analysis for identifying communities with overlapping proteins/genes. This would be a major step forward because it would require the inclusion of the hierarchy among the modules and as such, require fundamentally different algorithms.
